# High-intensity resistance exercise is not as effective as traditional high-intensity interval exercise for increasing the cardiorespiratory response and energy expenditure in recreationally active subjects

**DOI:** 10.1007/s00421-021-04849-4

**Published:** 2021-11-19

**Authors:** Laura Järvinen, Sofi Lundin Petersdotter, Thomas Chaillou

**Affiliations:** School of Health Sciences, Örebro University, 701 82 Örebro, Sweden

**Keywords:** Maximal oxygen uptake, Aerobic fitness, Resistance exercise, Heart rate, RPE, HIIT

## Abstract

**Purpose:**

Traditional high-intensity interval exercise (HIIE) highly stimulates the cardiorespiratory system and increases energy expenditure (EE) during exercise. High-intensity resistance exercise (HIRE) has become more popular in recreationally active subjects. The physiological responses to HIRE performed with light or moderate load is currently largely unknown. Here, we examined the effect of the type of interval exercise [HIRE at 40% (HIRE40) and 60% (HIRE60) 1-RM vs. traditional HIIE] on the cardiorespiratory response and EE during and after exercise.

**Methods:**

Fifteen recreationally active adults randomly completed traditional HIIE on an ergocyle, HIRE40 and HIRE60. The sessions consisted of two sets of ten 30-s intervals (power at 100% *V*O_2max_ during HIIE; maximal number of repetitions for 10 different free-weight exercises during HIRE40 and HIRE60) separated by 30-s active recovery periods. Gas exchange, heart rate (HR) and EE were assessed during and after exercise.

**Results:**

*V*O_2mean_, *V*O_2peak_, HR_mean_, the time spent above 90% *V*O_2max_ and HR_max_, and aerobic EE were lower in both HIRE sessions compared with HIIE (*P* < 0.05). Anaerobic glycolytic contribution to total exercise EE was higher in HIRE40 and HIRE60 compared with HIIE (*P* < 0.001). EE from excess post-exercise oxygen consumption (EPOC) was similar after the three sessions. Overall, similar cardiorespiratory responses and EE were found in HIRE40 and HIRE60.

**Conclusions:**

HIRE is not as effective as HIIE for increasing the cardiorespiratory response and EE during exercise, while EPOC remains similar in HIRE and HIIE. These parameters are not substantially different between HIRE40 and HIRE60.

## Introduction

Endurance exercise promotes the stimulation of the cardiorespiratory system and increases energy expenditure (EE) (Pinckard et al. 2019). In addition, EE remains elevated over the early period following endurance exercise due to high metabolic demands, which can be evaluated by the excess post-exercise oxygen consumption (EPOC) (Gaesser and Brooks 1984). Endurance training is highly recommended for improving cardiovascular fitness and improving body weight control (Skrypnik et al. 2015; Pinckard et al. 2019). Moderate-intensity continuous exercise and high-intensity interval exercise (HIIE) are the main forms of endurance exercise. HIIE appears to be equally, if not more effective, for improving maximum oxygen uptake (*V*O_2max_, a physiological determinant of cardiovascular fitness) as prolonged moderate-intensity continuous exercise, despite a limited training volume and time commitment (Gibala 2020). HIIE can also increase EPOC more importantly than isocaloric continuous exercise (Jung et al. 2019).

Traditional HIIE, commonly performed in individual endurance sports (e.g., running, cycling, rowing, etc.), is characterized by high-intensity intervals (i.e., performed near the power/speed eliciting *V*O_2max_), interspaced with periods of recovery. Several variables define a HIIE session, such as the intensity and duration of the working/recovery periods, and the number of sets (Buchheit and Laursen 2013). Over the last 20 years, numerous studies investigated the cardiorespiratory changes during HIIE sessions consisting of short working intervals (i.e., 30 s) performed at intensities near *V*O_2max_ (Billat et al. 2000; Thevenet et al. 2007a, b, 2008; Millet et al. 2003b; Rozenek et al. 2007; Rønnestad and Hansen 2016). These studies indicate that HIIE with short intervals are highly effective in eliciting prolonged times spent at a high percentage of *V*O_2max_ (i.e. ≥ 90% *V*O_2max_: T90%*V*O_2max_) or at a high percentage of maximal heart rate (HR) (i.e. ≥ 90% HR_max_: T90%HR_max_). HIIE with longer intervals could also be relevant for sustaining elevated T90%*V*O_2max_ and T90%HRmax, but whether it is more effective than HIIE with short intervals remains controversial (Rønnestad and Hansen 2016; Millet et al. 2003a). It has been proposed that the total time spent at high intensity is an important criterion to assess the effectiveness of a training program to improve *V*O_2max_ (Thevenet et al. 2007a; Midgley et al. 2006; Buchheit and Laursen 2013), and training interventions with traditional HIIE are very effective and time-efficient for developing *V*O_2max_ in healthy populations (Midgley et al. 2006; Wen et al. 2019; Gibala 2020). In addition, this training method highly increases EE during and after exercise (Skelly et al. 2014), and could have beneficial effects on body weight and body composition (Keating et al. 2017).

Recently, high-intensity resistance exercise (HIRE) and other types of high-intensity functional exercise have become more popular in recreationally active individuals (Claudino et al. 2018; Feito et al. 2018). HIRE consists of resistance exercises that combine numerous multiple-joint exercises performed at high intensity (usually defined as the maximal number of repetitions during a fixed period) with a load and in a circuit-type manner. High-intensity functional exercises are very attractive in recreationally active subjects because they can be modified to any fitness level, elicit varied muscle recruitment, and could be beneficial for improving muscle strength and muscle endurance (Munoz-Martinez et al. 2017; McRae et al. 2012; Myers et al. 2015; Feito et al. 2018). This training method also appears effective for developing *V*O_2max_ (Munoz-Martinez et al. 2017), but it is uncertain whether HIRE that recruit multiple and large muscle groups can elicit prolonged T90%*V*O_2max_ and T90%HR_max_, thereby imposing an optimal stimulus for improving *V*O_2max_ (Thevenet et al. 2007a; Midgley et al. 2006; Buchheit and Laursen 2013). Although the stimulation of the cardiorespiratory system may be lower during HIRE (i.e., combination of several resistance exercise bouts) compared with traditional HIIE (i.e., repetitions of intense aerobic exercises with a cyclic movement pattern), resulting in a lower EE from the aerobic system, these exercises could be more anaerobically demanding (Benito et al. 2016). It is, therefore, important to also consider the contribution of the anaerobic system to the total EE during exercise.

HIRE is generally practiced with loads between 20 and 80% 1-repetition maximum (1-RM) (Munoz-Martinez et al. 2017), and intervention studies showing an improvement of *V*O_2max_ usually included HIRE sessions performed with loads between 30 and 65% 1-RM (Munoz-Martinez et al. 2017). Due to the negative relationship between the number of repetitions and the % 1-RM in free-weight exercises (Shimano et al. 2006), it is evident that recreationally active subjects should perform HIRE with submaximal loads to accomplish a high number of repetitions and total working load, which may therefore highly solicit the cardiorespiratory system. To date, it is unclear whether HIRE executed at maximal effort (i.e., maximal number of repetitions during a fixed period) should be performed with light load (i.e., 40% 1-RM) or moderate load (60% 1-RM) to maximize T90%*V*O_2max_ and T90%HR_max_. Furthermore, it remains to be elucidated whether HIRE with light load and moderate load differently affect aerobic/anaerobic glycolytic exercise EE and EPOC.

Therefore, the aim of this study was to examine the effect of the type of interval exercise (HIRE with light load or moderate load vs. traditional HIIE) on the cardiorespiratory response (such as *V*O_2_, T90%*V*O_2max_, HR, T90%HR_max_) and EE during and after exercise in young recreationally active participants. We hypothesized that the cardiorespiratory response and aerobic exercise EE would be lower during the HIRE sessions than the HIIE session. In addition, we hypothesized that the anaerobic glycolysis exercise EE and EPOC would be greater during HIRE sessions than during HIIE session. Finally, these physiological parameters may be higher during HIRE with moderate load than HIRE with light load.

## Materials and methods

### Participants

Twenty-one recreationally active participants were initially recruited from two local fitness centers (Örebro, Sweden). The inclusion criteria were: aged between 20 and 40 years, being healthy and free of diagnosed cardiovascular and metabolic diseases, performing structured exercise training at least twice a week (with a total of light/moderate-intensity physical activity > 150 min per week), and having at least 1-year experience in resistance training. The exclusion criteria were: any disease, injury or another condition that could compromise the ability to perform the physical tests. Due to the withdrawal of five subjects and the exclusion of another one, 15 subjects (25.5 ± 3.3 years; 10 males and 5 females) were included in the study. The height, body mass, percentage of body fat (as described below), body mass index were 178.0 ± 10.8 cm, 81.4 ± 10.5 kg, 21.3 ± 7.4% and 25.8 ± 3.4 kg/m^2^, respectively. The participants were fully informed of the study procedures and a written informed consent was obtained from all participants. This study was performed in accordance with the declaration of Helsinki and was approved by the regional ethics committee of Uppsala (DNR 2018/321).

### Experimental design

The current study employed a randomized crossover design. The experiments consisted of seven visits to the laboratory or local fitness centers. All exercise sessions were separated by at least two days and the subjects did not perform any strenuous exercise at least 48 h prior to each exercise session. During the first visit, participants were familiarized with the equipment and anthropometric measurements and resting *V*O_2_ were determined. In visits two to three, 1-repetition maximum (1-RM) was determined for the ten exercises executed during the HIRE sessions (see description below). During the fourth visit, a maximal incremental cycling test was performed to determine *V*O_2max_ and the power eliciting *V*O_2max_ (*P*_max_). In visits five to seven, the participants performed three HIIE sessions in a random order. Each participant performed the physical tests and exercise sessions at a similar time of the day and was instructed to follow a similar diet before each test.

### Familiarization session

The familiarization session was performed in the morning after at least 12-h fasting. Body height was measured using standard procedures, and body mass and percentage of body fat were determined using bioelectrical impedance analysis (MC-780MA, Tanita Corporation, Japan). It is noteworthy that during the familiarization session, participants were only familiarized with the equipment used, but not with the physical tests and exercise sessions performed afterwards. For the determination of resting *V*O_2_, participants lied in a supine position for 15 min. *V*O_2_ was assessed during this period with a gas analyzer (Jaeger Oxycon Mobile, Vyaire Medical, USA), and resting *V*O_2_ calculated from the values obtained during the last 5 min was 3.35 ± 0.29 mL/kg/min.

### Selection of the weight exercise movements and determination of 1-RM

Ten exercises were selected for the HIRE sessions (Table 1), consisting of compound exercises that recruit multiple and large muscle groups. These exercises were executed with either a loaded barbell or dumbbells. Most of these exercises activated muscle groups from the lower limbs, trunk and upper limbs, as described in Table 1. The specific order of execution was chosen to limit the recruitment of similar muscle groups during successive exercises and to limit the accumulation of excessive fatigue that would prevent the proper execution of the movements. Since gas exchanges were measured during the entire HIRE sessions, it was important to select exercises that could be properly executed with the mobile gas analyzer. The participants wore the vest containing the device during all exercises, except during the last two exercises (i.e., chest press and hip thrust) due to the specific position.

**Table 1:** Weight exercise movements

Order	Movement	Description	Activation
1	Deadlift	Traditional BB deadlift from the floor	LL (+ + +), T (+ +), UL (+ +)
2	Step up	DB Step up to a bench	LL (+ + +), T ( +), UL (+ +)
3	Thruster	Front squat followed immediately by push press with DB	LL (+ + +), T (+ +), UL (+ + +)
4	Pendlay row	Pronated grip BB row from the floor	LL ( +), T (+ + +), UL (+ + +)
5	Reverse fly	Bent-over DB fly with slightly bent elbows	LL ( +), T (+ + +), UL (+ + +)
6	Romanian deadlift	Straight leg BB deadlift from hip to under knee position	LL (+ + +), T (+ +), UL (+ +)
7	Bent-over row	Supinated grip BB row from above the knee	LL ( +), T (+ + +), UL (+ + +)
8	Lunges	Forward lunge with DB	LL (+ + +), T (+ +), UL (+ +)
9	Chest press	Flat bench chest press with DB and feet up on the bench	LL (/), T (+ +), UL (+ + +)
10	Hip thrust	Thrust movement with BB placed on anterior pelvis	LL (+ + +), T (+ + +), UL (/)

Movements performed with a loaded barbell (BB) or with dumbbells (DB). Each exercise was performed during 30 s in both sets 1 and set 2, in the order presented in the left column

*LL* muscles of the lower limbs, *T* trunk muscles, *UL* muscles of the upper limbs,  +  light activation,  +  +  moderate activation,  +  +  +  high activation, / no or minor activation

1-RM for the 10 exercises of the HIRE sessions was estimated during tests performed at submaximal load (visits 2 and 3; 5 exercises per visit). Briefly, after a proper warm-up, subjects performed the tests with a submaximal load until failure (load estimated to be lifted 2–10 times). These tests (as well as the HIRE sessions) were performed at the local fitness center where participants had their membership. The Brzycki equation was used to estimate 1-RM (Mayhew et al. 1995). The load used during the HIRE sessions was calculated for each exercise according to 1-RM estimation (40% 1-RM during HIRE40 and 60% 1-RM during HIRE60).

### Maximal incremental cycling test

*V*O_2max_ was determined during a maximal incremental cycling test performed on a cycling ergometer (Monark LC6, Monark Exercise AB, Sweden). After a 5-min warm-up, power was increased by 20 W every minute until exhaustion. The subjects maintained a pedaling cadence above 70 rpm through the entire test. Expired gases were analyzed using a gas analyzer (Jaeger Oxycon Mobile, Vyaire Medical, USA) connected with JLAB software, and HR was recorded simultaneously using a coded transmitter belt (T31, Polar, Finland). Lactate concentration from fingertip blood sample ([La^−^]) was measured with a portable device (Lactate Plus, Cardioworld, Germany) three minutes after the completion of the test. The rate of perceived exertion (RPE, 6–20 Borg’s scale) was determined after each stage and at the end of the test.

Participants were considered to have reached their *V*O_2max_ when at least three of the five following criteria were met: (1) a plateau of *V*O_2_ (i.e., change ≤ 2,1 mL/kg/min), (2) a final respiratory exchange ratio (RER) ≥ 1.1, (3) maximum heart rate within 10 bpm of the age-predicted maximum [210-(0,65 × age)], (4) a post-test [La^−^] ≥ 8.0 mmol/L, and (5) RPE ≥ 18. *V*O_2max_, maximal ventilation (VE_max_), HR_max_ and RER_max_ were defined as the mean of the highest values obtained during six consecutive 5-s periods (30 s in total) and are presented in Table 2. Power at *V*O_2max_ (*P*_max_) was the lowest load that allowed *V*O_2max_ to be reached. If *V*O_2max_ was reached within the first 30 s of a specific stage, *P*_max_ was calculated as the power at this stage minus 10 W.

**Table 2:** Cardiorespiratory parameters during the maximal incremental cycling test and the three interval exercise sessions

	Incremental test	HIIE	HIRE40	HIRE60	*η*^*2*^/Kendall W
*V*O_2max/peak_ (mL/kg/min)	38.9 ± 7.1	41.8 ± 6.6$	33.7 ± 5.2$$ ***	34.4 ± 6.0$ ***	*0.734*
HR_max/peak_ (bpm)	188.5 ± 10.1	187.5 ± 12.6	180.3 ± 14.6	180.3 ± 10.5$*	0.364
VE_max/peak_ (L/min)	152.2 ± 44.1	132.0 ± 20.3	111.0 ± 28.1$$ **	117.1 ± 28.7$$ *	*0.598*
RER_max/peak_	1.31 ± 0.08	1.13 ± 0.08$$$	1.39 ± 0.13**	1.43 ± 0.13$**	*0.685*

Data are shown as mean ± SD (*N* = 11)

*VO*_*2*_ oxygen consumption, *HR* heart rate, *VE* ventilation, *RER* respiratory exchange ratio, *η*^*2*^ eta squared, highlighted in italic in the right column. Kendall W underlined in the right column

*P < 0.05, **P < 0.01, ***P < 0.001: significantly different compared with HIIE. $*P* < 0.05, $$*P* < 0.01, $$$*P* < 0.001: significantly different compared with the incremental test

### Interval exercises

The participants performed three exercise sessions in a random order and on three separate occasions. Each session began with a 10-min warm-up performed on a cycle ergometer at 60–70% maximum HR (determined during the maximal incremental test). The warm-up was followed by a 2-min transition period before the start of the session. Each exercise session consisted of two sets of ten 30-s working intervals interspaced with 30-s recovery periods. The two sets were separated by 4-min active walking recovery (i.e., between the last working interval of set 1 and the first working interval of set 2) at a self-controlled pace. This 4-min resting period was included between the two sets to limit extensive fatigue and to allow the recreationally active participants to complete the entire session (i.e., 10 min work). The scheme of the exercise sessions (i.e., duration of the intervals and recovery periods, number of exercise bouts per set, and number of sets) was identical for the three sessions. After the completion of the last working interval in set 2, the subjects were instructed to sit and rest for 12 min to evaluate EPOC and EE derived from EPOC during the early recovery phase. *V*O_2_ did not reach resting *V*O_2_ (which was assessed in a lying position) after 12 min. However, we decided to not extend the duration of the measurement to limit the discomfort after exercise and because *V*O_2_ was already stabilized at ~ 5–6 mL/kg/min after 12 min, a *V*O_2_ level used by others (Scott et al. 2011). Gas exchanges and HR were recorded during the warm-up and the exercise sessions, as described above. Blood lactate concentration was measured from fingertip at rest (before the warm-up) and three minutes after the two sets, and RPE (6–20 Borg’s scale) was determined directly after the two sets. These two parameters were evaluated to obtain indications of exercise intensity and perceived effort (Zinoubi et al. 2018; Tiggemann et al. 2010; Rønnestad et al. 2020). In addition, RER and VE were assessed during both sets.

The HIIE session was performed on a cycle ergometer (Monark LC6, Monark Exercise AB). The intensity of the 30-s working intervals was set at 100% *P*_max_ determined during the maximal incremental cycling test and the 30-s recovery intervals were performed at 50% *P*_max_. We selected this HIIE session because 30 s/30 s interval exercises at these intensities are well-tolerated by endurance-trained subjects and are effective in eliciting prolonged T90%*V*O_2max_ and T90%HR_max_ (Billat et al. 2000; Thevenet et al. 2007a, b, 2008; Millet et al. 2003b). As explained above, two sets of ten intervals interspaced with 4-min rest were used in our study (while 30 s intervals were performed until exhaustion by well-trained athletes in the later studies) to limit extensive fatigue and to allow our participants to achieve 10 min at high intensity. Indeed, results from an unpublished pilot study performed in our laboratory showed that recreationally active subjects (*N* = 7; 24.3 ± 2.7 years) were able to complete this HIIE session and maintain high percentages of *V*O_2max_ and HR_max_. Despite these preliminary tests, 4 of our 15 recreationally active participants were unable to complete the two sets of HIIE due to severe exhaustion. For this reason, these participants performed a second HIIE session (at least 48 h later) that consisted of two sets of ten 30-s working interval (95% *P*_max_) interspaced with 30-s recovery intervals (50% *P*_max_).

The two HIRE sessions consisted of two sets of ten 30-s working intervals (intensity described below) separated by 30-s recovery periods (time used to put down the loaded barbell or dumbbells, walk and be ready for the next exercise). Each exercise movement (described in Table 1) was performed during one 30-s working interval period in both sets. The order of the ten exercises is presented in Table 1 and the choice of the exercises was justified above. The exercises were executed at a load corresponding to 40% of 1-RM during the HIRE40 session, and 60% of 1-RM during the HIRE60 session. Our pilot study revealed that participants could perform on average 10–12 repetitions at 60% 1-RM during 30-s working intervals. Selecting a higher % 1-RM would have limited the number of repetitions performed and thus would have certainly provoked important breaks during the working intervals. In opposite, selecting 40% 1-RM was a good option to increase the number of repetitions performed while maintaining a high total load lifted (see Table 5). The load used during the HIRE sessions was rounded to the closest available weight, which was never more than 1.2 kg below or above the calculated load. The subjects were familiarized with the exercises and the order of the exercises before starting the HIRE sessions. The participants were instructed to perform the 30-s working intervals of HIRE sessions at their maximal sustainable work intensity, aiming to execute the highest number of repetitions of each movement. The number of repetitions and the load lifted during each set was calculated to evaluate the cumulated work of HIRE40 and HIRE60 sessions.

Due to the specific nature of our exercise protocols, the performed work of the exercise sessions could not be matched for total work. Instead, our approach was based on the matching for the perceived effort (i.e. RPE) assessed directly after exercise, which applies more to real world training (Rønnestad et al. 2020). We validated this approach with our specific setting during a pilot experiment (as described above). This experiment showed that RPE was 19.0 ± 0.8 after a HIIE (same protocol as in the current study) and 19.4 ± 1.4 after HIRE60 (same protocol as in the current study, except that five exercise movements were executed twice in each set) (*P* = 0.41), indicating that the perceived effort was similar in both sessions.

### Analysis of cardiorespiratory parameters, energy expenditure and EPOC during and after the exercise sessions

The cardiorespiratory values were averaged over a 5-s period to determine *V*O_2mean_, *V*O_2peak_, VE_peak_, HR_mean_, HR_peak_, RER_mean_ and RER_peak_. The gas analyzer used in this study (Jaeger Oxycon Mobile, Vyaire Medical) is considered to be a reliable device, with coefficients of variation from 1.6 to 5.8% for *V*O_2_ and RER over a range of intensities (submaximal to maximal) (Rosdahl et al. 2010). *V*O_2peak_, HR_peak_, VE_peak_ and RER_peak_ were defined as the mean of the highest values obtained during six consecutive 5-s periods. *V*O_2mean_ and HR_mean_ were calculated in both sets 1 and 2, during the entire interval exercise period (i.e. average of sets 1 and 2, excluding the recovery period between the two sets). In Table 3, *V*O_2peak_ and *V*O_2mean_ were expressed as a percentage of *V*O_2max_ (determined during the maximal incremental test) and HR_peak_ and HR_mean_ were expressed as a percentage of HR_max_ (determined during the maximal incremental test). T90%*V*O_2max_ and T90%HR_max_ were calculated during each set and during the entire exercise session (i.e. cumulated time spent during sets 1 and 2).

**Table 3:** Cardiorespiratory parameters during the three interval exercise sessions

	HIIE	HIRE40	HIRE60	*η*^*2*^/Kendall W
*V*O_2mean_ (% *V*O_2max_)				
Set 1	89.0 ± 4.5	62.5 ± 7.5*** #	66.8 ± 7.8***	*0.900*
Set 2	95.6 ± 6.5	64.2 ± 7.4***	64.8 ± 6.1***	*0.950*
Average	92.3 ± 5.2	63.3 ± 7.3***	65.8 ± 6.8***	*0.948*
Rest	36.4 ± 5.2	36.2 ± 6.2	38.8 ± 5.3	*0.223*
*V*O_2peak_ (% *V*O_2max_)				
Set 1	103.4 ± 6.6	84.4 ± 11.3***	88.4 ± 10.1***	*0.753*
Set 2	107.8 ± 6.3	85.9 ± 9.2***	86.5 ± 8.1***	*0.859*
T90%*V*O_2max_ (s)				
Set 1	319.5 ± 113.4	22.3 ± 29.7***	40.0 ± 55.1*	0.903
Set 2	465.5 ± 91.3	25.0 ± 40.0***	20.00 ± 25.0**	0.814
Total	785.0 ± 191.6	47.3 ± 63.4***	60.0 ± 76.0*	0.917
HR_mean_ (% HR_max_)				
Set 1	89.6 ± 4.5	84.3 ± 6.2*	87.1 ± 3.7*	*0.471*
Set 2	94.6 ± 3.8	88.7 ± 6.3*	89.0 ± 3.0**	0.504
Average	92.1 ± 4.2	86.5 ± 6.1**	88.0 ± 3.1*	0.504
Rest	73.4 ± 6.6	72.2 ± 8.1	74.4 ± 4.5	0.078
HR_peak_ (% HR_max_)				
Set 1	95.7 ± 3.7	93.1 ± 6.0	94.2 ± 3.6	0.174
Set 2	99.4 ± 3.3	95.6 ± 5.4	95.4 ± 3.4*	0.370
T90%HR_max_ (s)				
Set 1	352.3 ± 178.2	158.6 ± 141.9*	240.5 ± 161.8*	*0.470*
Set 2	512.7 ± 56.6	340.9 ± 158.3**	282.3 ± 170.0**	*0.557*
Total	865.0 ± 232.9	499.5 ± 276.7**	522.7 ± 291.4***	*0.569*

Data are shown as mean ± SD (*N* = 11)

*VO*_*2*_ oxygen consumption, *T90%VO*_*2max*_ time above 90% VO_2max_,. *HR* heart rate, *T90%HRmax* time above 90% HR_max_,. *η*^*2*^ eta squared, highlighted in italic in the right column. Kendall W underlined in the right column. Rest represents the resting period between the two sets

*P < 0.05, **P < 0.01, ***P < 0.001: significantly different compared with HIIE. #*P* < 0.05: significantly different compared with HIRE60

The VO_2_ values were averaged over a 30-s period to determine aerobic EE and EPOC. Three different caloric equivalents were used for the calculation of EE. The caloric equivalent was set at 4.85 kcal/L of O_2_ during the warm-up, and at 5.05 kcal/L of O_2_ during the interval exercise period (sets 1 and 2) (Schaun et al. 2017). The caloric equivalent was set at 4.64 kcal/L O_2_ during the between-set recovery period and the 12-min post-exercise period to exclude rapid glycolytic ATP resynthesis (Benito et al. 2016). Indeed, exercise anaerobic glycolytic EE was estimated using the O_2_ equivalent of blood lactate accumulation, as previously described (di Prampero and Ferretti 1999; Benito et al. 2016). During the three interval exercise sessions, Δ[La^−^] was calculated by subtracting resting values from peak [La^−^] reached 3 min after exercise. Δ[La^−^] was then converted to O_2_ equivalent values as 3 mL O_2_/kg body mass/mmol of Δ[La^−^] (Benito et al. 2016). Exercise anaerobic glycolytic EE was calculated using the caloric equivalent of 5.05 kcal/L of O_2_. Total exercise EE was defined as the sum of aerobic EE and anaerobic glycolytic EE during sets 1 and 2. EPOC was calculated every min during the 12-min post-exercise period using the following formula: EPOC (L/min) = *V*O_2_ post-exercise (L/min) − resting VO_2_ (L/min). EPOC was then converted to EE as 1 L of O_2_ = 4.64 kcal to exclude rapid glycolytic ATP resynthesis as part of the conversion of O_2_ uptake into EE (Benito et al. 2016). EPOC represents the O_2_ consumption for aerobic ATP turnover and an estimation of the fast resynthesis of ATP and phosphocreatine (PC) stores (Scott et al. 2011).

### Statistical analysis

**Fig. 1:**
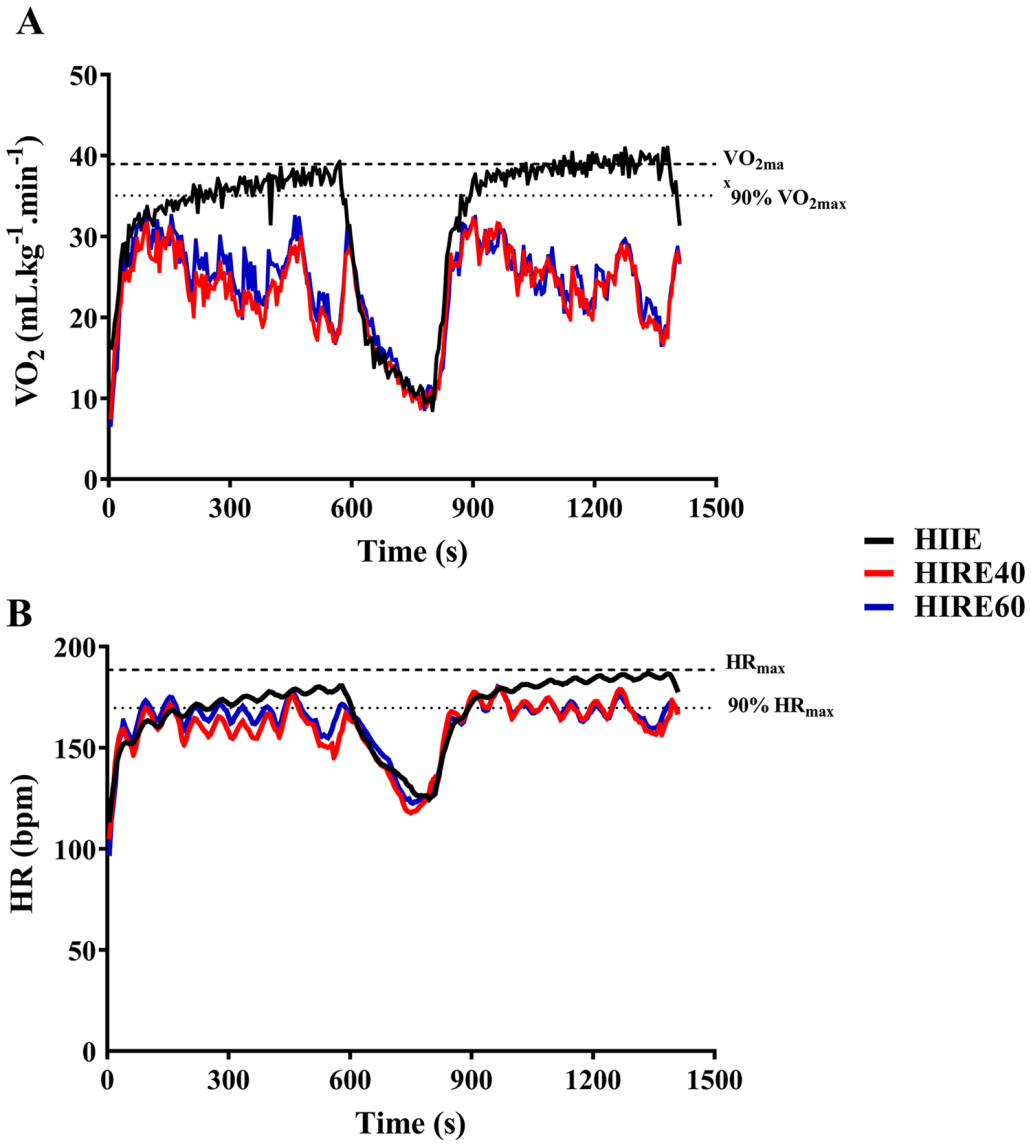
VO_2_ (**A**) and HR (**B**) during the three interval exercise sessions. Data are the mean values obtained from 11 participants.

**Fig. 2:**
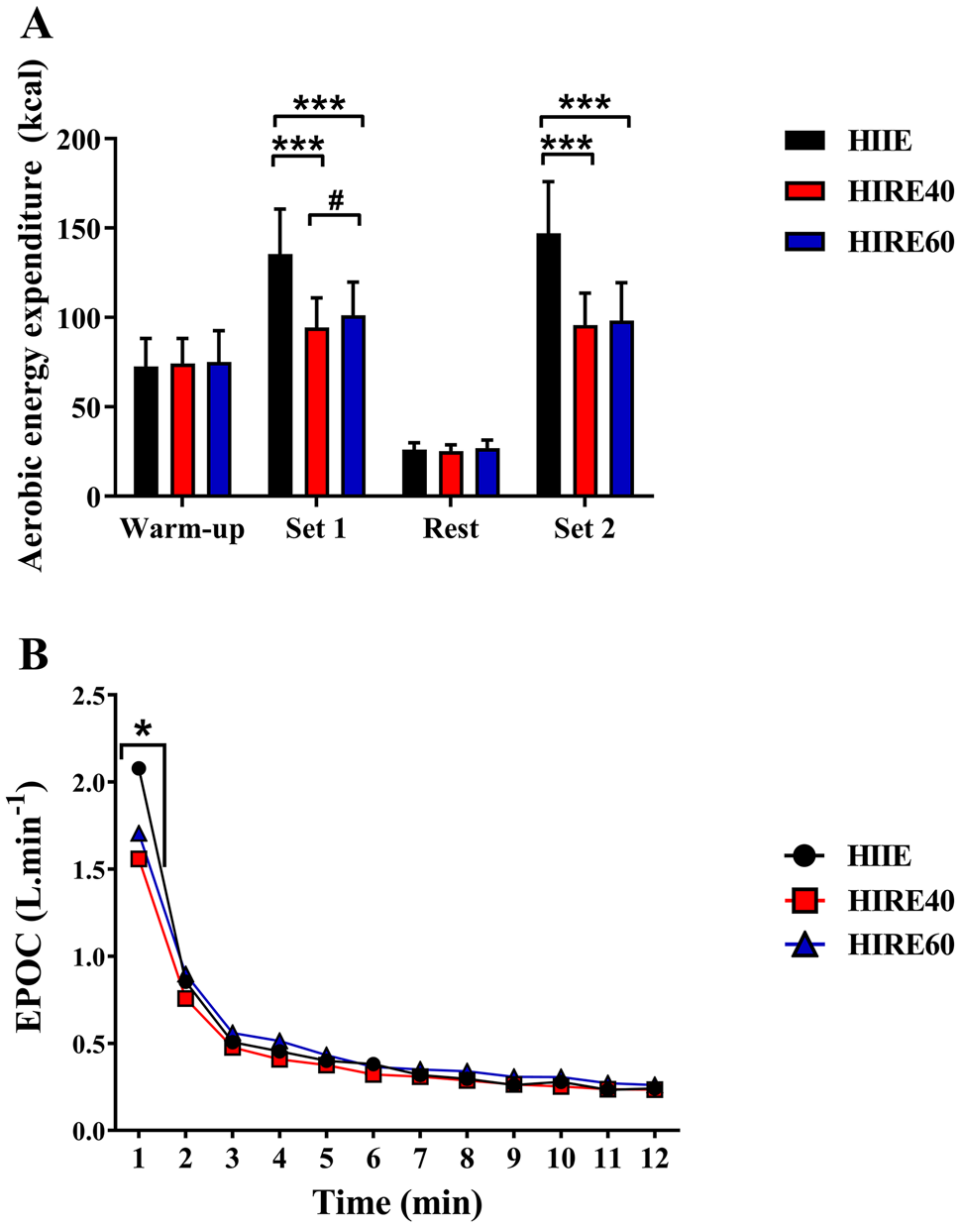
Energy expenditure during the exercise session (**A**) and excess post-exercise oxygen consumption (EPOC) during the 12-min post-exercise period (**B**) for the three interval exercise sessions. **A** Data are shown as mean ± SD. *N* = 14 during the warm-up, *N* = 15 during Set 1, *N* = 14 during rest, *N* = 12 during Set 2. ***P < 0.001, significantly different compared with HIIE. #*P* < 0.05, significant difference between HIRE40 and HIRE60. **B** Data are the mean values obtained from 11 participants. *P < 0.05, significant difference between HIIE and HIRE40

Data are presented as mean ± standard deviation (SD). Statistical analyses were performed with Graphpad Prism (version 8.3) and SPSS Statistics (version 27; for analysis of effect size only). Shapiro–Wilk tests were used to check normality assumption. Skewed data were log-transformed and normal distribution was assessed again before selecting the appropriate parametric or non-parametric statistical tests. The following parameters were analyzed using one-way repeated measures analysis of variance (RM ANOVA): VO_2max/peak_ (log-transformed), VE_max/peak_, RER_max/peak_, VO_2mean_ [set 1 (log-transformed), set 2, average of sets 1 and 2, and rest], VO_2peak_ (sets 1 and 2), HR_mean_ (set 1), T90%HR_max_ (all time periods), blood lactate concentration (sets 1 and 2), RER (sets 1 and 2), RPE (set 1), EE (all measurements) and EPOC. When a significant main effect was observed, Bonferroni post hoc tests were used to evaluate the differences between the three exercise sessions. The following parameters were analyzed using Friedman’s test: HR_max/peak_, HR_mean_ (set 2, average of sets 1 and 2, and rest), HR_peak_ (sets 1 and 2), T90%VO_2max_ (all time periods), and RPE (set 2). When a significant main effect was observed, Dunn’s multiple comparisons tests were used to evaluate the differences between the three exercise sessions. Two-tailed paired t tests were used to compare the number of repetitions and the load lifted between HIRE40 and HIRE60, and to compare the between-set changes in load lifted between HIRE40 and HIRE60. The α-level of significance was set at *P* < 0.05. Eta squared (η^2^) (for parametric tests) and Kendall W (for Friedman’s tests) were determined to estimate the effect size. Effects sizes were classified as small (η^2^: from 0.02 to 0.13; Kendall W: from 0.1 to 0.3), moderate (η^2^: from 0.13 to 0.26; Kendall W: from 0.3 to 0.5) and large (η^2^: above 0.26; Kendall W: above 0.5). A power analysis was performed from a pilot experiment (see the description above) using G*Power (version 3.1.9.4). The desired power was set to 0.8 and alpha was set at 0.05. Power analyses performed on T90VO_2max_ (average of sets 1 and 2) and *V*O_2mean_ (average of sets 1 and 2) gave a sample size of 13 and 4 subjects, respectively. Fifteen subjects were included in the study, however, due to technical issues during the experiments (e.g., missing values of cardiorespiratory parameters and missing blood samples), the total number of participants included in the final analyses was between 11 and 15. The number of participants for each specific variable is presented in Table 5, and in the legend of Tables 2, 3 and 4 and Figs. 1 and 2.

**Table 4:** Energy expenditure from the aerobic and anaerobic glycolytic metabolisms during exercise and from excess post-exercise oxygen consumption (EPOC) during the 12-min post-exercise period

	HIIE	HIRE40	HIRE60	*η*^*2*^
Energy expenditure (kcal)				
Aerobic	282.3 ± 55.1	187.9 ± 38.4***	198.8 ± 41.7***	*0.920*
Anaerobic glycolytic	11.2 ± 2.8	13.5 ± 4.8	14.7 ± 3.2**	*0.353*
Energy expenditure (%)				
Aerobic	96.1 ± 0.9	93.5 ± 1.3***	93.0 ± 1.1***	*0.714*
Anaerobic glycolytic	3.9 ± 1.0	6.5 ± 1.6***	7.0 ± 1.3***	*0.714*
EPOC (kcal)	29.2 ± 5.8	25.4 ± 6.7	29.2 ± 7.2	*0.155*

Data are shown as mean ± SD (*N* = 12 during exercise; *N* = 11 for EPOC). *η*^*2*^ eta squared, highlighted in italic in the right column. Energy expenditure during exercise represents the sum of the energy expenditure during sets 1 and 2

**P < 0.01, ***P < 0.001: significantly different compared with HIIE

## Results

### Comparison of the cardiorespiratory parameters during the maximal incremental cycling test and the three interval exercises

The highest values of *V*O_2_, HR, VE and RER during the maximal incremental cycling test (maximal values) and the interval exercise sessions (peak values) are presented in Table 2. *V*O_2max_ (incremental test) was significantly higher than *V*O_2peak_ observed during HIRE40 (15%, *P* = 0.008) and HIRE60 (13%, *P* = 0.022), while *V*O_2max_ was significantly lower than *V*O_2peak_ observed during HIIE (− 7%, *P* = 0.013). HR_max_ (incremental test) was significantly higher than HR_peak_ observed during HIRE60 (5%, *P* = 0.039). VE_max_ (incremental test) was significantly higher than VE_peak_ observed during HIRE40 and HIRE60 (~ 25%, *P* < 0.01). RER_max_ (incremental test) was significantly higher than RER_peak_ observed during HIIE (14%, *P* < 0.001), while RER_max_ was significantly lower than RER_peak_ observed during HIRE60 (− 8%, *P* = 0.034).

### Cardiorespiratory response during the exercise sessions

The changes in *V*O_2_ during the three exercise sessions are described in Fig. 1A. During HIIE, *V*O_2_ rapidly increased and was on average higher than 90% *V*O_2max_ after approximately 4 and 1 min in set 1 and set 2, respectively. During HIRE40 and HIRE60, *V*O_2_ rapidly increased over the first min of each set, and then highly fluctuated at a relatively moderate level. Overall, changes in *V*O_2_ were similar during HIRE40 and HIRE60. *V*O_2_ similarly decreased between the two sets for the three HIIE sessions.

*V*O_2mean_ measured during HIRE40 and HIRE60 was substantially lower (~−25 to 30%) than during HIIE (*P* < 0.001; Table 3). During set 1, *V*O_2mean_ was slightly lower (− 6%, *P* = 0.032) in HIRE40 than in HIRE60. These differences were not found between HIRE40 and HIRE60 during set 2 and during the whole session (average of sets 1 and 2). *V*O_2mean_ was not different between the three sessions during the between-set recovery period (rest) (Table 3). *V*O_2peak_ measured during HIRE40 and HIRE60 was lower (~−15 to 20%) than during HIIE (*P* < 0.001), with no significant differences found between HIRE40 and HIRE60 (Tables 2 and 3). *V*O_2_ was maintained above 90% VO_2max_ over a prolonged period during both sets 1 and 2 for HIIE (Table 3). T90%*V*O_2max_ was very limited (≤ 1 min in total) during both HIRE40 and HIRE60, and was significantly lower than during HIIE (*P* < 0.001 and *P* = 0.032, respectively; Table 3). T90%*V*O_2max_ was not significantly different between HIRE40 and HIRE60. During set 1, all subjects reached 90% *V*O_2max_ in HIIE, while they were 6/11 and 9/11 to reach this level during HIRE40 and HIRE60, respectively. During set 2, all participants also reached 90% *V*O_2max_ in HIIE, while they were 5/11 and 7/11 to reach 90%*V*O_2max_ during HIRE40 and HIRE60, respectively.

The changes in HR during the three exercise sessions are described in Fig. 1B. During HIIE, HR rapidly increased and was on average higher than 90% HR_max_ after approximately 4 and 1 min 30 s in set 1 and set 2, respectively. During HIRE40 and HIRE60, HR rapidly increased over the first 3 min of each set, and then fluctuated during the remaining period of each set. Overall, the changes in HR were similar during HIRE40 and HIRE60. HR similarly decreased between sets 1 and 2 for the three sessions.

HR_mean_ measured during HIRE40 and HIRE60 was lower (~−5%) than during HIIE (*P* < 0.05), with no significant differences found between HIRE40 and HIRE60 (Table 3). HR_mean_ was not different between the three sessions during the between-set recovery period (rest) (Table 3). During set 1, HR_peak_ was not significantly different between HIIE, HIRE40 and HIRE60 (Table 3). During set 2, HR_peak_ was slightly lower in HIRE60 compared with HIIE (*P* = 0.032), while no other significant differences were observed for this parameter. During sets 1 and 2, T90%HR_max_ was significantly lower for both HIRE40 and HIRE60 compared with HIIE (*P* < 0.05; Table 3). In total, T90%HR_max_ was ~ 40% reduced during HIRE40 and HIRE60 compared with HIIE (*P* = 0.003 and *P* < 0.001, respectively), while no significant differences were observed between HIRE40 and HIRE60. During both sets 1 and 2, all subjects reached 90% HR_max_ in HIIE, while they were 10/11 to reach this level during HIRE40 and HIRE60.

### Energy expenditure

Aerobic EE calculated during the three exercise sessions (including the warm-up, set 1, recovery between sets and set 2) is presented in Fig. 2A. Aerobic EE during the warm-up and the in-between-set recovery period were similar for the three sessions. During sets 1 and 2, aerobic EE was significantly lower in HIRE40 and HIRE60 compared with HIIE (set 1: ~ −25 to 30%, *P* < 0.001; η^2^ = 0.859; set 2: ~ 35%, *P* < 0.001, η^2^ = 0.934). Aerobic EE was slightly lower in HIRE40 than HIRE60, but only during set 1 (− 7%, *P* = 0.045).

Table 4 depicts the aerobic and anaerobic glycolytic EE during the exercise sessions (set 1 + set 2). As shown in Fig. 2A, exercise aerobic EE was significantly lower in HIRE40 and HIRE60 compared with HIIE (*P* < 0.001) while no significant differences were found between the two HIRE sessions. Exercise anaerobic glycolytic EE was higher in HIRE60 than in HIIE (*P* = 0.007), whereas it was not different in HIRE40 compared with the two other interval exercises. Anaerobic glycolytic contribution to total exercise EE was higher in HIRE40 and HIRE60 compared with HIIE (*P* < 0.001), while the opposite result was found for the aerobic system (*P* < 0.001).

EPOC assessed during the 12-min post-exercise period is presented in Fig. 2B. EPOC rapidly decreased during the first min following the end of the exercises, and then remained relatively stable (~ 0.25 L/min) after 10 min. EPOC was only significantly different between the exercise sessions at 1 min post-exercise (main effect: *P* = 0.018; η^2^ = 0.410), being significantly lower in HIRE40 compared with HIIE (*P* = 0.031). EE derived from EPOC (i.e., EE for aerobic ATP turnover and for the fast resynthesis of ATP/PC stores) was not significantly different between the exercise sessions (Table 4).

### Blood lactate concentration, RER, RPE and load lifted

Blood lactate concentration was similar at baseline for the three exercise sessions (HIIE: 1.0 ± 0.4 mmol/L; HIRE40: 1.1 ± 0.6 mmol/L; HIRE60: 1.0 ± 0.5 mmol/L). After sets 1 and 2, blood lactate concentration was neither significantly different between HIIE and HIRE40, nor between HIRE40 and HIRE60, while it was significantly higher in HIRE60 than in HIIE (*P* < 0.001; Table 5).

**Table 5:** Blood lactate concentration, rate of perceived exertion and load lifted

	HIIE	HIRE40	HIRE60	*η*^*2*^/Kendall W	*N*
Blood lactate concentration (mM)					
Set 1	8.3 ± 2.5	10.0 ± 2.4	11.6 ± 3.1***	*0.398*	13
Set 2	9.8 ± 2.6	11.7 ± 2.9	12.3 ± 3.3**	*0.300*	15
RER_mean_					
Set 1	1.05 ± 0.06	1.15 ± 0.07**	1.17 ± 0.07***	*0.609*	15
Set 2	0.95 ± 0.03	1.04 ± 0.05**	1.01 ± 0.04***	*0.628*	12
RPE (6–20)					
Set 1	17.3 ± 1.0	15.7 ± 1.9* ##	17.4 ± 1.2	*0.986*	15
Set 2	19.0 ± 0.8	17.8 ± 1.9	19.1 ± 1.0	0.240	
Load lifted (kg)					
Set 1	/	5247 ± 1390#	5734 ± 1059	*0.253*	15
Set 2	/	4792 ± 1110	4530 ± 1078	*0.107*	
Total	/	10,045 ± 2371	10,264 ± 1996	*0.029*	
Number of repetitions					
Set 1	/	157.0 ± 21.4###	117.4 ± 18.4	*0.832*	15
Set 2	/	147.4 ± 28.7###	93.1 ± 18.6	*0.855*	
Total	/	303.4 ± 44.6###	210.5 ± 34.0	*0.916*	

Data are shown as mean ± SD

*RER* respiratory exchange ratio, *RPE* rate of perceived exertion, *η*^*2*^ eta squared, highlighted in italic in the right column

*P < 0.05, **P < 0.01, ***P < 0.001: significantly different compared with HIIE. #*P* < 0.05, ##*P* < 0.01, ###*P* < 0.001: significantly different compared with HIRE60. Kendall W underlined in the right column

RER_peak_ was significantly higher in HIRE40 and HIRE60 compared with HIIE (*P* < 0.01; Table 2). RER_mean_ was significantly higher in HIRE40 and HIRE60 compared with HIIE during both set 1 (*P* = 0.003 and *P* < 0.001, respectively) and set 2 (*P* = 0.002 and *P* < 0.001, respectively), whereas no differences were observed between the two HIRE sessions (Table 5).

After set 1, RPE was significantly lower in HIRE40 compared to HIRE60 (*P* = 0.003) and HIIE (*P* = 0.015), while no differences were found between HIRE60 and HIIE (Table 5). No significant differences between the three exercise sessions were found for RPE after set 2.

The total number of repetitions was significantly lower during HIRE40 than during HIRE60 (*P* < 0.001; Table 4). The load lifted was significantly higher for HIRE60 than for HIRE40 during set 1 (9%, *P* = 0.047; Table 5), while no differences were found between the two HIRE sessions during set 2 or in total. The decrease in load lifted during set 2 compared to set 1 was more pronounced for HIRE60 than for HIRE40 (− 20.7 ± 11.8% vs. −6.9 ± 13.0%, *P* = 0.004; η^2^ = 0.454).

## Discussion

Physiological responses to interval exercise are determined by numerous parameters, including the duration and intensity of the exercise and recovery periods, as well as the type of exercise (e.g., cycling, running, resistance exercise, etc.) (MacInnis and Gibala 2017). In this study, we examined the effect of the type of interval exercise (HIRE40 and HIRE60 vs. HIIE) on the cardiorespiratory response, and EE during and after exercise. In accordance with our hypothesis, we showed that the cardiorespiratory response to exercise was markedly reduced during HIRE40 and HIRE60 compared with HIIE, as demonstrated by lower *V*O_2mean_, *V*O_2peak_, T90%*V*O_2max_, HR_mean_, T90%HR_max_ and VE_peak_. Aerobic exercise EE was also highly reduced in HIRE60 and HIRE40 compared with HIIE, while the anaerobic glycolytic contribution to total exercise EE was higher in HIRE than HIIE sessions. EPOC EE during the early recovery phase was similar in the three exercise sessions. In contrast to our hypothesis, the cardiorespiratory response, EE during exercise and EPOC were not substantially different between HIRE40 and HIRE60.

Most studies comparing acute physiological responses between different interval exercises focused on exercise modalities where protocols were matched for total work, or where objective intensity (i.e., power or speed) was controlled during interval exercises repeated over a fixed period or until exhaustion (Billat et al. 2000; Thevenet et al. 2007a, b, 2008; Rozenek et al. 2007; Smilios et al. 2018; Tucker et al. 2015). In the present study, the three exercise sessions had identical durations. However, exercise/recovery intensity was based on the power during HIIE, while the power could not be controlled and assessed during the two HIRE sessions due to their specific nature. Thus, working bouts of the HIRE sessions were executed at maximal sustainable work intensity and the intensity was assessed from the perceived effort (i.e., RPE) directly after the session. This method, which has been recently used by others (Rønnestad et al. 2020), constitutes a relevant approach applied to real world training. As shown in our pilot study (see in the method section), the present results confirmed that RPE was not different between HIIE and HIRE sessions directly after exercise, indicating that the perceived effort was comparable between the three interval exercises.

One of the main finding of this study is that T90%*V*O_2max_ was on average maintained during the major part of HIIE, a result in agreement with previous studies focusing on 30 s/30 s HIIE (Thevenet et al. 2007a, 2008), while T90%*V*O_2max_ was on average ≤ 1 min during HIRE40 and HIRE60. This result is consistent with the lower *V*O_2mean_ observed during HIRE40 and HIRE60 compared with HIIE. Several factors may explain this difference in cardiorespiratory response. First, although *V*O_2peak_ remained lower during HIRE40 and HIRE60 compared with HIIE, numerous participants reached 90% *V*O_2max_ during the two HIRE sessions, but this high *V*O_2_ level could not be sustained. The highest *V*O_2_ were observed when exercise movements highly recruited lower limb muscles, while low VO_2_ levels were found when exercises mostly mobilized upper limbs and/or trunk muscles (see Table 1; Fig. 1A). This observation is consistent with the idea that the level of *V*O_2_ measured during intense exercise is determined by the amount of muscle mass being recruited (Ballor et al. 1987; Orr et al. 2013). Second, differences in stroke volume and in heart rate (to a smaller extent; see Table 3) have probably impaired the increase in cardiac output during HIRE sessions compared with HIIE, thereby limiting oxygen consumption. Although not assessed in this study, the potential lower stroke volume during HIRE sessions may result from high-intensity muscle contractions (which can reduce preload and increased afterload) (Miles et al. 1987) and Valsalva maneuver associated with heavy lifting (which increases intrathoracic and intraabdominal pressures and impairs venous return) (Lentini et al. 1993). Other factors may be involved in the reduced oxygen consumption observed during HIRE compared with HIIE, including a greater peripheral vasoconstriction (and thus a lower arteriovenous O_2_ difference) due to a potential higher sympathetic activity during HIRE sessions, the recruitment of fiber types with different metabolic properties (which could affect muscle O_2_ extraction) (Cardinale et al. 2019), the nature of muscle contractions, as well as the feature of the 30-s recovery periods. Indeed, these recovery periods could not be identically performed during the HIRE sessions (transition to move to the next exercise station) and HIIE (cycling at 50% P_max_). Apparent reductions of VO_2_ and HR were found during the 30-s recovery periods of HIRE sessions (see Fig. 1), which certainly have affected the overall cardiorespiratory response. Finally, blood lactate concentration was significantly higher for HIRE60 than HIIE (values observed in HIRE40 being between those found in HIRE60 and HIIE), and RER_mean_ and RER_peak_ were higher in both HIRE sessions (being > 1.0 for RER_mean_ and > 1.35 for RER_peak_) compared with HIIE. These findings indicate a larger stimulation of the anaerobic glycolytic system (as also shown in Table 4) during HIRE than HIIE sessions, which certainly contributes to explain the differences in cardiorespiratory response between HIRE and HIIE.

An intriguing result of this study is that *V*O_2peak_ during HIIE was higher than VO_2max_. The question as to whether *V*O_2max_ measured during a maximal incremental test is a real maximal value has been previously raised (Schaun 2017). To our knowledge, this phenomenon has not been reported in studies with a similar protocol (i.e., HIIE including a fixed number of repetitions). However, other studies using HIIE performed until exhaustion have reported higher VO_2_ values during HIIE than during a maximal incremental test (Billat et al. 2000; Chang et al. 2020).

Aerobic EE have previously been assessed during interval exercise sessions consisting of various types of exercises (Schaun et al. 2018, 2017; Islam et al. 2018; Benito et al. 2016; Falcone et al. 2015), but to our knowledge, no studies have compared exercise EE between traditional HIIE and HIRE. Similarly to the cardiorespiratory response, aerobic EE was substantially lower during HIRE sessions compared with HIIE. In addition, the anaerobic glycolytic contribution to total exercise EE was larger during HIRE40 and HIRE60 than during HIIE, and this contribution is in the same range as that reported by others during HIRE (Benito et al. 2016). However, the higher activation of the anaerobic glycolytic system during HIRE sessions cannot offset the limited aerobic EE during exercise, and therefore total EE remains clearly reduced during HIRE sessions compared with HIIE session. Small differences for EPOC were only observed during the first minute of post-exercise recovery (see Fig. 2B) due to the larger level of VO_2_ at the end of the HIIE session. However, EPOC EE following exercise was not significantly different between the three exercise sessions. Since early EPOC provide indications of the fast resynthesis of ATP/PC stores (Scott 2006; Scott et al. 2011), our results suggest that the phosphagens metabolism was unlikely differentially affected between the three exercise sessions.

In contrast to our second hypothesis, no marked differences in cardiorespiratory response and energy expenditure were found between HIRE40 and HIRE60. Interestingly, VO_2mean_ and aerobic EE were slightly lower in HIRE40 than HIRE60 during set 1, a result that corroborates with the differences observed for RPE and the load lifted during set 1. The reduced load lifted during set 2 compared with set 1 was more important in HIRE60 than HIRE40, but the cumulated load lifted was similar during both sessions. Therefore, HIRE60 appears to induce more fatigue and is more demanding than HIRE40 during set 1, while the perceived effort and total work achieved at the end of both sessions are similar. This latter result most likely explains the absence of differences of cardiorespiratory response and energy expenditure between the two HIRE sessions. The contribution of anaerobic glycolysis to exercise EE, blood lactate concentration during exercise, and EPOC were not different between HIRE40 and HIRE60, which further indicate that overall, HIRE40 and HIRE60 induced a similar physiological response, at least for the variables analyzed in this study.

## Study limitations

A few limitations should be highlighted from this study. First, we intentionally selected exercises soliciting various muscle groups to limit the accumulation of excessive fatigue that characterized HIRE. However, the choice of exercises was limited since the movements were executed with a mobile gas analyzer and using free weights. Although other exercises may have been more advantageous to reach and maintain high levels of *V*O_2_, it is unlikely that those exercises (i.e., movements mostly recruiting lower limb muscles) could have been executed at maximal intensity during 10 bouts of 30 s by recreationally active subjects. Another limitation is the loss of data of cardiorespiratory parameters during and after exercise, which was due to technical issues with our mobile gas analyzer. Our sample size (11 to 15 subjects included in the final analyses) was close to or higher than the estimated sample size calculated from our power analysis (see statistical analysis), and the effect sizes observed in this study were large for most variables. This indicates that our statistical analysis was certainly not underpowered. Furthermore, the anaerobic glycolytic EE was estimated using the O_2_ equivalent of blood lactate accumulation, a method widely used (Benito et al. 2016; di Prampero and Ferretti 1999; Bertuzzi et al. 2010). Although informative, it may not precisely estimate the contribution of the anaerobic glycolytic system in our study due to (1) our exercise protocol (e.g., alternance of intervals, intensity not controlled during HIRE sessions), (2) our experimental design ([La-] should have been measured at several time-points during exercise and rest) and 3) other limitations (e.g., the metabolic demand of active muscles may not be accurately estimated by analyzing whole-body physiological responses) (Bertuzzi et al. 2010). Finally, although ATP/PC component constitutes a part of EPOC (Scott 2006), some limitations regarding the use of EPOC has been reported (Green and Dawson 1993). Future works should develop better methods to estimate the anaerobic contribution during interval exercises.

## Practical implications and perspectives

Our results clearly demonstrate that different types of interval exercise can induce various physiological responses in recreationally active subjects. Therefore, a specific interval exercise session should be chosen in accordance with the training purpose. If the main objective is to perform a short and intense session that highly solicits the cardiorespiratory system and markedly increases EE, HIIE using large muscle groups (e.g., cycling, running, rowing, etc.) should be advised. HIRE should be rather encouraged for strength and conditioning training, allowing to strengthen different muscle groups while moderately activating the cardiorespiratory system and increasing EE. However, some recommendations could be provided to optimize T90%VO_2max_ during HIRE. First, selected exercise movements should recruit major muscle groups and/or engage several muscle groups together (e.g., lower limb with upper limb/trunk muscles), while avoiding the execution of successive exercises that mobilize similar muscle groups to limit the accumulation of excessive fatigue. Second, exercise bouts should be performed at maximal intensity (i.e., execution of the highest number of repetitions for each movement) while maintaining a proper technique to avoid any risks of injury. Since the intensity was not sustainable during the set 2 of HIRE60 (see Table 4), it may be more appropriate to perform HIRE at 40% 1-RM, at least in recreationally active subjects. Finally, it may also be relevant to shorten the duration of the sets to limit the accumulation of fatigue (e.g., 6 min instead of 10 min) while increasing the number of sets per session. This option could be helpful to progressively increase the working time over a training intervention. Although our results indicate that HIRE does not induce an optimal stimulus on the cardiorespiratory system for improving *V*O_2max_, recent studies have demonstrated that high-intensity functional training enables to develop it (Munoz-Martinez et al. 2017; McRae et al. 2012). Future studies should compare the improvement of *V*O_2max_ after a training intervention including HIIE or HIRE.

## Conclusion

Our study demonstrates that HIRE is not as effective as HIIE for increasing the cardiorespiratory response and EE during exercise in recreationally active subjects. However, EPOC EE during the early recovery phase is similar in HIRE and HIIE sessions. Our results also show that the cardiorespiratory response and EE during exercise, and EPOC are not substantially different between HIRE performed with low or moderate load.

## Data Availability

On request.

## Abbreviations

1-RM: 1-Repetition maximum
ATP: Adenosine triphosphate
EE: Energy expenditure
EPOC: Excess post-exercise oxygen consumption
HIIE: High-intensity interval exercise
HIRE: High-intensity resistance exercise
HIRE40: High-intensity resistance exercise at 40% 1-repetition maximum
HIRE60: High-intensity resistance exercise at 60% 1-repetition maximum
HR: Heart rate
HR_max_: Maximal heart rate
HR_mean_: Mean heart rate
HR_peak_: Peak heart rate
PC: Phosphocreatine
Pmax: Power eliciting maximal oxygen uptake
RM ANOVA: One-way repeated measures analysis of variance
RPE: Rate of perceived exertion
RER: Respiratory exchange ratio
RER_max_: Maximal respiratory exchange ratio
RER_mean_: Mean respiratory exchange ratio
RER_peak_: Peak respiratory exchange ratio
T90%HR_max_: Time spent above 90% maximal heart rate
T90%*V*O_2max_: Time spent above 90% maximal oxygen uptake
VE: Ventilation
VE_max_: Maximal ventilation
VE_peak_: Peak ventilation
*V*O_2_: Oxygen uptake
*V*O_2max_: Maximal oxygen uptake
*V*O_2mean_: Mean oxygen uptake
*V*O_2peak_: Peak oxygen uptake
η^2^: Eta squared
[La^−^]: Blood lactate concentration

## References

[CR1] Ballor DL, Becque MD, Katch VL (1987). Metabolic responses during hydraulic resistance exercise. Med Sci Sports Exerc.

[CR2] Benito PJ, Alvarez-Sanchez M, Diaz V, Morencos E, Peinado AB, Cupeiro R, Maffulli N (2016). Cardiovascular Fitness and Energy Expenditure Response during a Combined Aerobic and Circuit Weight Training Protocol. PLoS ONE.

[CR3] Bertuzzi RC, Franchini E, Ugrinowitsch C, Kokubun E, Lima-Silva AE, Pires FO, Nakamura FY, Kiss MA (2010). Predicting MAOD using only a supramaximal exhaustive test. Int J Sports Med.

[CR4] Billat VL, Slawinski J, Bocquet V, Demarle A, Lafitte L, Chassaing P, Koralsztein JP (2000). Intermittent runs at the velocity associated with maximal oxygen uptake enables subjects to remain at maximal oxygen uptake for a longer time than intense but submaximal runs. Eur J Appl Physiol.

[CR5] Buchheit M, Laursen PB (2013). High-intensity interval training, solutions to the programming puzzle: Part I: cardiopulmonary emphasis. Sports Med.

[CR6] Cardinale DA, Larsen FJ, Jensen-Urstad M, Rullman E, Søndergaard H, Morales-Alamo D, Ekblom B, Calbet JAL, Boushel R (2019). Muscle mass and inspired oxygen influence oxygen extraction at maximal exercise: role of mitochondrial oxygen affinity. Acta Physiol (oxf).

[CR7] Chang SC, Adami A, Lin HC, Lin YC, Chen CPC, Fu TC, Hsu CC, Huang SC (2020). Relationship between maximal incremental and high-intensity interval exercise performance in elite athletes. PLoS ONE.

[CR8] Claudino JG, Gabbett TJ, Bourgeois F, Souza HS, Miranda RC, Mezêncio B, Soncin R, Cardoso Filho CA, Bottaro M, Hernandez AJ, Amadio AC, Serrão JC (2018). CrossFit overview: systematic review and meta-analysis. Sports Med Open.

[CR9] di Prampero PE, Ferretti G (1999). The energetics of anaerobic muscle metabolism: a reappraisal of older and recent concepts. Respir Physiol.

[CR10] Falcone PH, Tai CY, Carson LR, Joy JM, Mosman MM, McCann TR, Crona KP, Kim MP, Moon JR (2015). Caloric expenditure of aerobic, resistance, or combined high-intensity interval training using a hydraulic resistance system in healthy men. J Strength Cond Res.

[CR11] Feito Y, Heinrich KM, Butcher SJ, Poston WSC (2018). High-intensity functional training (HIFT): definition and research implications for improved fitness. Sports (basel).

[CR12] Gaesser GA, Brooks GA (1984). Metabolic bases of excess post-exercise oxygen consumption: a review. Med Sci Sports Exerc.

[CR13] Gibala MJ (2020). Physiological basis of interval training for performance enhancement. Exp Physiol.

[CR14] Green S, Dawson B (1993). Measurement of anaerobic capacities in humans. Definitions, limitations and unsolved problems. Sports Med.

[CR15] Islam H, Townsend LK, Hazell TJ (2018). Excess postexercise oxygen consumption and fat utilization following submaximal continuous and supramaximal interval running. Res Q Exerc Sport.

[CR16] Jung WS, Hwang H, Kim J, Park HY, Lim K (2019). Effect of interval exercise versus continuous exercise on excess post-exercise oxygen consumption during energy-homogenized exercise on a cycle ergometer. J Exerc Nutrition Biochem.

[CR17] Keating SE, Johnson NA, Mielke GI, Coombes JS (2017). A systematic review and meta-analysis of interval training versus moderate-intensity continuous training on body adiposity. Obes Rev.

[CR18] Lentini AC, McKelvie RS, McCartney N, Tomlinson CW, MacDougall JD (1993). Left ventricular response in healthy young men during heavy-intensity weight-lifting exercise. J Appl Physiol.

[CR19] MacInnis MJ, Gibala MJ (2017). Physiological adaptations to interval training and the role of exercise intensity. J Physiol.

[CR20] Mayhew JL, Prinster JL, Ware JS, Zimmer DL, Arabas JR, Bemben MG (1995). Muscular endurance repetitions to predict bench press strength in men of different training levels. J Sports Med Phys Fitness.

[CR21] McRae G, Payne A, Zelt JG, Scribbans TD, Jung ME, Little JP, Gurd BJ (2012). Extremely low volume, whole-body aerobic-resistance training improves aerobic fitness and muscular endurance in females. Appl Physiol Nutr Metab.

[CR22] Midgley AW, McNaughton LR, Wilkinson M (2006). Is there an optimal training intensity for enhancing the maximal oxygen uptake of distance runners?: empirical research findings, current opinions, physiological rationale and practical recommendations. Sports Med.

[CR23] Miles DS, Owens JJ, Golden JC, Gotshall RW (1987). Central and peripheral hemodynamics during maximal leg extension exercise. Eur J Appl Physiol Occup Physiol.

[CR24] Millet GP, Candau R, Fattori P, Bignet F, Varray A (2003). VO2 responses to different intermittent runs at velocity associated with VO2max. Can J Appl Physiol.

[CR25] Millet GP, Libicz S, Borrani F, Fattori P, Bignet F, Candau R (2003). Effects of increased intensity of intermittent training in runners with differing VO2 kinetics. Eur J Appl Physiol.

[CR26] Munoz-Martinez FA, Rubio-Arias JA, Ramos-Campo DJ, Alcaraz PE (2017). Effectiveness of resistance circuit-based training for maximum oxygen uptake and upper-body one-repetition maximum improvements: a systematic review and meta-analysis. Sports Med.

[CR27] Myers TR, Schneider MG, Schmale MS, Hazell TJ (2015). Whole-body aerobic resistance training circuit improves aerobic fitness and muscle strength in sedentary young females. J Strength Cond Res.

[CR28] Orr JL, Williamson P, Anderson W, Ross R, McCafferty S, Fettes P (2013). Cardiopulmonary exercise testing: arm crank vs cycle ergometry. Anaesthesia.

[CR29] Pinckard K, Baskin KK, Stanford KI (2019). Effects of exercise to improve cardiovascular health. Front Cardiovasc Med.

[CR30] Rønnestad BR, Hansen J (2016). Optimizing interval training at power output associated with peak oxygen uptake in well-trained cyclists. J Strength Cond Res.

[CR31] Rønnestad BR, Hansen J, Nygaard H, Lundby C (2020). Superior performance improvements in elite cyclists following short-interval vs effort-matched long-interval training. Scand J Med Sci Sports.

[CR32] Rosdahl H, Gullstrand L, Salier-Eriksson J, Johansson P, Schantz P (2010). Evaluation of the Oxycon Mobile metabolic system against the Douglas bag method. Eur J Appl Physiol.

[CR33] Rozenek R, Funato K, Kubo J, Hoshikawa M, Matsuo A (2007). Physiological responses to interval training sessions at velocities associated with VO2max. J Strength Cond Res.

[CR34] Schaun GZ (2017). The maximal oxygen uptake verification phase: a light at the end of the tunnel?. Sports Med Open.

[CR35] Schaun GZ, Alberton CL, Ribeiro DO, Pinto SS (2017). Acute effects of high-intensity interval training and moderate-intensity continuous training sessions on cardiorespiratory parameters in healthy young men. Eur J Appl Physiol.

[CR36] Schaun GZ, Pinto SS, Praia ABC, Alberton CL (2018). Energy expenditure and EPOC between water-based high-intensity interval training and moderate-intensity continuous training sessions in healthy women. J Sports Sci.

[CR37] Scott CB (2006). Contribution of blood lactate to the energy expenditure of weight training. J Strength Cond Res.

[CR38] Scott CB, Leighton BH, Ahearn KJ, McManus JJ (2011). Aerobic, anaerobic, and excess postexercise oxygen consumption energy expenditure of muscular endurance and strength: 1-set of bench press to muscular fatigue. J Strength Cond Res.

[CR39] Shimano T, Kraemer WJ, Spiering BA, Volek JS, Hatfield DL, Silvestre R, Vingren JL, Fragala MS, Maresh CM, Fleck SJ, Newton RU, Spreuwenberg LP, Häkkinen K (2006). Relationship between the number of repetitions and selected percentages of one repetition maximum in free weight exercises in trained and untrained men. J Strength Cond Res.

[CR40] Skelly LE, Andrews PC, Gillen JB, Martin BJ, Percival ME, Gibala MJ (2014). High-intensity interval exercise induces 24-h energy expenditure similar to traditional endurance exercise despite reduced time commitment. Appl Physiol Nutr Metab.

[CR41] Skrypnik D, Bogdański P, Mądry E, Karolkiewicz J, Ratajczak M, Kryściak J, Pupek-Musialik D, Walkowiak J (2015). Effects of endurance and endurance strength training on body composition and physical capacity in women with abdominal obesity. Obes Facts.

[CR42] Smilios I, Myrkos A, Zafeiridis A, Toubekis A, Spassis A, Tokmakidis SP (2018). The effects of recovery duration during high-intensity interval exercise on time spent at high rates of oxygen consumption, oxygen kinetics, and blood lactate. J Strength Cond Res.

[CR43] Thevenet D, Tardieu-Berger M, Berthoin S, Prioux J (2007). Influence of recovery mode (passive vs active) on time spent at maximal oxygen uptake during an intermittent session in young and endurance-trained athletes. Eur J Appl Physiol.

[CR44] Thevenet D, Tardieu M, Zouhal H, Jacob C, Abderrahman BA, Prioux J (2007). Influence of exercise intensity on time spent at high percentage of maximal oxygen uptake during an intermittent session in young endurance-trained athletes. Eur J Appl Physiol.

[CR45] Thevenet D, Leclair E, Tardieu-Berger M, Berthoin S, Regueme S, Prioux J (2008). Influence of recovery intensity on time spent at maximal oxygen uptake during an intermittent session in young, endurance-trained athletes. J Sports Sci.

[CR46] Tiggemann CL, Korzenowski AL, Brentano MA, Tartaruga MP, Alberton CL, Kruel LF (2010). Perceived exertion in different strength exercise loads in sedentary, active, and trained adults. J Strength Cond Res.

[CR47] Tucker WJ, Sawyer BJ, Jarrett CL, Bhammar DM, Gaesser GA (2015). Physiological responses to high-intensity interval exercise differing in interval duration. J Strength Cond Res.

[CR48] Wen D, Utesch T, Wu J, Robertson S, Liu J, Hu G, Chen H (2019). Effects of different protocols of high intensity interval training for VO(2)max improvements in adults: a meta-analysis of randomised controlled trials. J Sci Med Sport.

[CR49] Zinoubi B, Zbidi S, Vandewalle H, Chamari K, Driss T (2018). Relationships between rating of perceived exertion, heart rate and blood lactate during continuous and alternated-intensity cycling exercises. Biol Sport.

